# The relationship between competitive anxiety and athlete burnout in college athlete: the mediating roles of competence and autonomy

**DOI:** 10.1186/s40359-024-01888-2

**Published:** 2024-07-17

**Authors:** Linjie Yang, Zhiwen Zhang, Jinrui Zhang, Arsaythamby Veloo

**Affiliations:** 1 Sports Training College, Chengdu Sport University, Chengdu, 610041 Sichuan China; 2 Faculty of Social Sciences and Liberal Arts, UCSI University, Kuala Lum pur, 56000 Malaysia; 3 Faculty of Sports and Health, Chongqing Electronic Information College, Chongqing, 400900 China; 4 Department of Physical Education, Neijiang Normal University, Neijiang, 641100 Sichuan China; 5 School of Education and Modern Languages, Universiti Utara Malaysia, Sintok Bukit Kayu, Hitam, 06010 Malaysia; 6 College of Physical Education and Health, Guangxi Normal University, Guilin, China 541006

**Keywords:** Athlete burnout, Sport anxiety, Self-determination theory, Competence, Autonomy, College athlete

## Abstract

**Background:**

In the cognitive-affective model of athlete burnout, anxiety is a key physiological response to stress that influences the development of burnout in athletes. Despite its importance, there has been little research on the relationship between competitive anxiety and athlete burnout, particularly regarding the mediating mechanisms. This study aimed to explore the relationship between competitive anxiety and athlete burnout, with a focus on the mediating role of general need satisfaction from self-determination theory.

**Methods:**

The current study employed a cross-sectional design involving 618 college athletes (354 females, mean age 20.57 years), comprising 303 participants in individual sports and 315 in team sports. These participants completed the Sport Anxiety Scale-2 (SAS-2), the Athlete Burnout Questionnaire (ABQ), and the Basic Psychological Need Satisfaction Scale in General (BPNSS-G) online. Subsequently, correlation, regression, and mediation analyses were conducted using SPSS and JASP to examine the relationships between the variables.

**Results:**

Regression results indicated that somatic anxiety (beta = 0.116, *t* = 2.21, *p* = 0.028) and concentration disruption (beta = 0.259, *t* = 5.35, *p* < 0.001) in competitive anxiety were positively correlated with athlete burnout. Worry in competitive anxiety was negatively correlated with competence (beta =-0.149, *t*=-2.70, *p* = 0.007) and autonomy (beta =-0.179, *t*=-3.25, *p* = 0.001) in needs satisfaction. Additionally, the regression results found that competence (beta =-0.178, *t*=-3.39, *p* = 0.001) and Autonomy (beta =-0.208, *t*=-4.17, *p* < 0.001) were negatively associated with athlete burnout. Mediation analyses revealed significant direct effects in the relationship between somatic anxiety (Effect = 0.116, *p* = 0.026) along with concentration disruption (Effect = 0.259, *p* < 0.001) and athlete burnout. In the indirect effect, worry (Effect = 0.071, *p* = 0.002) as well as concentration disruption (Effect = 0.082, *p* < 0.001) had significant effects in relation to athlete burnout, respectively.

**Conclusions:**

Overall, the current study found that somatic anxiety and concentration disruption in competitive anxiety are related to athlete burnout. Moreover, competence and autonomy in need satisfaction mediated the relationship between competitive anxiety (worry and concentration disruption) and athlete burnout. The findings of this study not only shed further light on the relationship between competitive anxiety and athlete burnout but also provide theoretical insights into the mediating mechanisms of this relationship.

## Introduction

Burnout is a syndrome characterized by emotional exhaustion, a reduced sense of accomplishment, and depersonalization [1]. Burnout doesn’t only impact our work; it can also have a detrimental effect on our well-being [2–4]. In sport psychology, Raedeke (1997) defined athlete burnout as a psychological syndrome comprising emotional and physical exhaustion, reduced sense of accomplishment, and sport devaluation [5]. Emotional and physical exhaustion is characterized by extreme fatigue and emotional depletion, which are associated with the intense demands of training and competition. Reduced sense of accomplishment reflects negative evaluations of one’s athletic skills and abilities. Lastly, sport devaluation occurs reflects athletes who no longer care about the sport or their performance, losing interest and desire for the sport [5, 6]. Athlete burnout is an important topic that has garnered considerable attention from researchers [7–9], due to its association with a range of detrimental psychophysiological behavioral outcomes.

Specifically, previous research has found that higher athlete burnout (i.e., higher scores on the three subscales of the Athlete Burnout Questionnaire, ABQ) is associated with lower performance and a higher risk of dropping out among elite handball players aged 14 to 18 [10]. In addition, studies in elite winter athletes have found that emotional exhaustion in athlete burnout is positively correlated with parental expectations and criticism and negatively correlated with the pursuit of high personal standards [11]. Reduced accomplishment is positively correlated with worry about mistakes and doubts about actions and negatively correlated with perceived ability and the pursuit of high standards. Sport devaluation is positively correlated with ego orientation and doubts about actions and negatively correlated with perceived ability. Furthermore, the prevalence of burnout among college athletes has shown an increasing trend over the past 20 years [12, 13]. Therefore, it is urgent to study the antecedent variables affecting athlete burnout and to theoretically examine the mediating mechanisms between these antecedent variables and athlete burnout.

To better explain the process of burnout in athletes, Smith (1986) proposed the cognitive-affective model of athletic burnout, depicting the dynamic relationship between stress and burnout through four components: situational, cognitive, physiological, and behavioral [14]. An athlete may face an imbalance between environmental demands and personal resources, such as competing against a strong opponent. When needs are unmet, negative emotions like anxiety, guilt, and anger may arise. In this situation, the athlete assesses the demands, resources, consequences, and personal significance of these consequences. Physiological responses, such as anxiety, occur when the assessment indicates a threat of harm. These physiological responses lead to behavioral and coping responses that may include athlete burnout. Meanwhile, individual differences in motivation and personality influence all four components of the burnout process. Additionally, sport competition involves both competitive pressure and social evaluative factors, leading athletes to often experience sport-specific trait anxiety before or during competition. Therefore, this study aims to investigate the relationship between competitive anxiety and athlete burnout, with self-determination theory (SDT), as a mediating factor, to provide additional insights into this relationship.

## Theoretical framework

### The relationship between competitive anxiety and athlete burnout

Anxiety is an emotion characterized by unpleasant tension, worrying thoughts, and corresponding physical symptoms [15]. In sports, the competitive pressure to perform often triggers sports-specific anxiety, encompassing somatic arousal, worry, and concentration disruption [16, 17]. Where somatic arousal includes various indicators of autonomic arousal centered on the stomach and muscles, worry encompasses the fear of poor performance and its negative consequences, and concentration disruption includes the difficulty of focusing on task-related cues [17]. According to the cognitive-affective model of athletic burnout [14], athletes may face an imbalance between environmental demands and personal resources. When these demands are unmet, it can result in anxiety, guilt, anger, and self-restraint. Additionally, athletes make a cognitive appraisal of the situation (e.g., what the potential outcome means to them). If this appraisal indicates a threat, physiological responses such as anxiety will occur, ultimately leading to a behavioral burnout response [14]. Therefore, researchers have studied athletic competitive anxiety and burnout in athletes using various measurement tools.

For example, a study by Raedeke and Smith (2001) found that somatic anxiety, worry, and concentration disruption in competitive anxiety (as measured by the Sport Anxiety Scale, SAS) among college athletes were significantly and positively correlated with emotional and physical exhaustion, reduced sense of accomplishment, and sport devaluation in athlete burnout [6]. Another study divided female ice hockey and soccer athletes into facilitative and debilitative anxiety groups based on their scores on the Competitive State Anxiety Inventory-2D and found that the debilitative anxiety group had significantly higher burnout scores (as measured by the Burnout Inventory for Athletes) than the facilitative group [18]. The researchers further found in a study of 106 athletes that the debilitative anxiety group scored significantly higher than the facilitative group on reduced sense of accomplishment (as measured by the ABQ), while there were no significant differences in emotional and physical exhaustion and sport devaluation [19]. A study of 368 Korean college athletes found that somatic anxiety, worry, and concentration disruption in competition anxiety (measured by the SAS-2) were significantly and positively correlated with emotional and physical exhaustion and sport devaluation in athlete burnout, while no significant correlation was found with reduced sense of accomplishment [20].

However, a recent study of Chinese college athletes found that worry and concentration disruption were significantly associated with the three dimensions of burnout: emotional/physical exhaustion, reduced sense of accomplishment, and sport devaluation [21]. Overall, the studies mentioned above indicate that competitive anxiety is significantly and positively correlated with athlete burnout. However, inconsistent findings emerge when examining specific dimensions of athlete burnout. This raises the question of whether competitive anxiety correlates only with certain dimensions of athlete burnout and not with others. Therefore, the first goal of the current study was to examine the relationship between competitive anxiety and different dimensions of athlete burnout using a cross-sectional study of a large sample of college athletes.

### The mediating role of basic need satisfaction in the relationship between competitive anxiety and athlete burnout

The symptoms of athlete burnout are often described as a loss of motivation, which can include staying away from friends and colleagues or even withdrawing from sport. This also suggests a meaningful relationship between athletic burnout and motivation [7]. To better understand athlete burnout and its antecedents, researchers have begun to apply the self-determination theory, a motivation theory, in the field of athlete burnout research [22]. The SDT is a macro-theory of human motivation, personality, and well-being, consisting of six sub-theories widely used in various fields [23]. The most widely used sub-theory in athlete burnout research is basic psychological needs theory, which presents the three basic needs of autonomy, relatedness, and competence, exploring their relationship with psychological health/well-being [24]. Under the appropriate conditions, these three basic psychological needs can guide people to adopt behaviors that are more competent, vital, and socially integrated [24].

Researchers have used both cross-sectional and longitudinal designs across various samples to find that all three types of need fulfillment in SDT show varying degrees of negative correlation with athlete burnout [25–28]. For example, a cross-sectional study by Perreault et al. (2007) found that satisfaction of the three needs—autonomy, competence, and relatedness—was negatively correlated with reduced sense of accomplishment, emotional and physical exhaustion, and sport devaluation, respectively [25]. A subsequent cross-sectional study found that these needs in elite athletes (from 51 different sports, averaging 9.5 years of practice) were significantly negatively correlated with reduced sense of accomplishment, emotional and physical exhaustion, and sport devaluation [27]. Another study by Amorose et al. (2009) used a longitudinal design to measure need satisfaction and athlete burnout among adolescent female volleyball players 1–2 weeks before the start of the season and at the end of the season. The results showed that, after controlling for the three end-of-season need satisfaction scores, all three pre-season need satisfactions scores (competence, autonomy, and relatedness) negatively predicted post-season athlete burnout [26].

Notably, some researchers have examined the mediating role of need satisfaction in SDT and motivation in the relationship between antecedent variables and athlete burnout. For example, a study by Eleanor and Joan (2010) investigated the relationship between perceived social environment and both positive and negative affect, as well as the mediating role of need satisfaction among dancers (mean duration of dance practice was 12.12 years) [29]. The results found that ego-involving climates (i.e., intraindividual competition and rivalry among participants) were negatively correlated with need satisfaction in SDT for competence and relatedness, which in turn were negatively correlated with negative affect and positively correlated with positive affect. Additionally, another study examined the relationship between perfectionism and athlete burnout among junior athletes using a longitudinal design based on SDT [30]. It was found that autonomous motivation mediated the negative correlation between perfectionistic strivings and athlete burnout at the within-subject level.

Meanwhile, need satisfaction in SDT was found to be negatively correlated with anxiety [31, 32], and it mediated the relationship between anxiety and outcome variables [33, 34]. For example, one study investigated the relationship between social physique anxiety, need satisfaction in SDT, and physical activity among college students. The results showed that social physique anxiety was significantly and negatively related to need satisfaction, and that need satisfaction (competence and relatedness) mediated the relationship between social physique anxiety and physical activity [34]. Additionally, another study found that somatic anxiety, worry, and concentration disruption in competitive anxiety were positively correlated with all three dimensions of athlete burnout [35]. It also found that threat perception and challenge perception in cognitive appraisal mediated the relationship between competitive anxiety and athlete burnout.

### The current study

Overall, the above findings highlight the relationship between competitive anxiety and athlete burnout, along with the mediating role of need satisfaction in athlete burnout and its antecedent variables. Building on these findings, the current study will examine the relationship between competitive and athlete burnout, focusing on the potential mediating role of the three types of need satisfaction in SDT. Based on the findings of previous studies [6, 20, 22], we hypothesized that competitive anxiety would positively correlate with athlete burnout (Hypothesis 1). Secondly, previous research has found that autonomous motivation mediates the relationship between perfectionistic strivings and athlete burnout [30], and need satisfaction mediates the relationship between social physique anxiety and physical activity [34]. Therefore, we propose Hypothesis 2: need satisfaction in SDT mediates the relationship between competitive anxiety and athlete burnout.

## Method

### Participants

This study ultimately included 618 college athletes (354 males and 264 females) from multiple universities who voluntarily participated via the Tencent questionnaire platform (similar to Amazon Turk), taking approximately 10 min to complete the survey. The mean age of the participants was 20.57 years, with a standard deviation of 3.05. Among these participants, 351 were first-year students, 158 were second-year students, 26 were third-year students, 6 were fourth-year students, and 77 were graduate students. The participants were involved in various sports, including basketball, football, volleyball, table tennis, badminton, athletics, dance, gymnastics, and swimming, among others. Based on their involvement, participants were categorized into individual (e.g., swimming, *n* = 303) and team sports (e.g., basketball, *n* = 315). Previous research has found that when testing for mediating effects with a power of 0.8, a minimum of 558 participants are required in a percentile bootstrap estimate when both the $\:\alpha\:$ path and $\:\beta\:$ path have a small effect size (0.14) [36]. The sample size of the current study is 618, indicating that the current sample size has good statistical power.

To ensure data quality, we included a careful answer check item in the questionnaire. Consequently, in the subsequent data analysis, we excluded participants who answered this item incorrectly, as well as those who completed the questionnaire within three minutes, removing an additional 91 participants. Furthermore, we excluded an additional 67 participants as outliers during the preliminary analyses based on the box plot results for each subscale score. Participants accessed and completed the questionnaire using their mobile phones during physical education sessions. During this process, they first read and signed the informed consent form, then reviewed the instructions, and finally completed the questionnaire. Participation in the survey was voluntary, and all participants received appropriate compensation in the form of course credits. All participants provided informed consent by reading and signing a consent form before taking part, and the study received approval from the ethics committee of the local university.

### Measures

#### The sport anxiety Scale-2 (SAS-2)

The SAS-2 was selected for this study to assess participants’ competitive anxiety because multidimensional anxiety theory suggests that competitive anxiety can occur at both the somatic and cognitive levels [37]. The SAS-2 is a competitive trait anxiety scale based on this theory, used to assess both somatic and cognitive symptoms of sport performance anxiety [17]. It assesses anxiety levels before or during a competition and includes three subscales: somatic anxiety, worry, and concentration disruption, with five items per subscale. Somatic anxiety assesses physiological elements of hyper-activation, such as stomach tension (e.g., “Before or while I compete in sports, I feel tense in my stomach”). Worry, on the other hand, assesses concerns related to poor performance (e.g., “Before or while I compete in sports, I worry that I won’t play well”). Concentration disruption measures difficulties in focusing on relevant aspects of the competitive activity (e.g., “Before or while I compete in sports, it is hard to concentrate on the game”). Participants rated each item on a four-point Likert scale, ranging from 1 (not at all) to 4 (very much). Higher scores indicate higher competition anxiety, while lower scores indicate lower anxiety of that type. The study utilized the Chinese version of the SAS-2, which has undergone translation and psychometric testing in prior study [38]. Cronbach’s reliability coefficients for Somatic anxiety, Worry, and Concentration disruption were 0.81, 0.89, and 0.78, respectively, with a total scale reliability coefficient of 0.92.

#### Athlete burnout questionnaire (ABQ)

The ABQ developed by Raedeke and Smith [6], is a multidimensional tool designed to measure burnout in athletes. It comprises 15 items that assess three dimensions: emotional and physical exhaustion, reduced achievement, and sport devaluation, with five items for each dimension.

Participants rated each item on a five-point Likert scale, ranging from 1 (almost never) to 5 (almost always) (e.g., “I feel successful at sports”). The Chinese version of the ABQ, which has been translated and tested for its psychometric properties in previous study [21], was used in this study. The Cronbach reliability coefficients for the three subscales were 0.71 (reduced sense of accomplishment), 0.86 (emotional and physical exhaustion), and 0.79 (sport devaluation). The total reliability of the ABQ (15 items) was 0.88.

#### Basic psychological need satisfaction scale-in general (BPNSS-G)

The BPNSS-G developed by Deci and Ryan [24] based on SDT, assesses the satisfaction of overall basic needs in an individual’s life. Comprising 21 items, the scale measures the three needs of competence, autonomy, and relatedness. In the current study, we used the translated and psychometrically validated Chinese version of the BPNSS-G from a previous study [39]. Considering that the BPNSS-G measures various aspects of a person’s life, the Chinese version has been appropriately amended to address the current research focus. Specifically, “in physical activities” has been added to some items, or “in daily life” has been changed to “in physical activities”. For example, “Often, in sports activities, I do not feel very competent” (competence); “In sports activities, I really like the people I interact with” (relatedness); and “In sports activities, I generally feel free to express my ideas and opinions” (autonomy). This version includes 19 items, with participants responding on a 7-point Likert scale (1 = not at all, 7 = very true). It included 6 items each for autonomy and competence, and 7 items for relatedness. The reliability coefficients in this study were 0.71 (competence), 0.73 (autonomy), 0.77 (relatedness), and 0.86 (total scale) based on Cronbach’s alpha.

### Statistical analysis

In this study, all data were analyzed using IBM SPSS 22.0 and JASP 0.18.3.0. JASP is a freely available and widely used statistical analysis software that provides a range of tools for various statistical analyses. We first performed an outlier analysis on the data and then conducted a preliminary analysis after excluding these outliers. This included tests for reliability, skewness, kurtosis, mean, standard deviation, and normality for each variable. Then, the correlations between the variables were calculated. Next, regression analyses were conducted to investigate the mediating role of the three need satisfactions between competitive anxiety and athlete burnout, based on the mediation effect test procedure proposed in previous study [40]. Finally, the effects and 95% confidence intervals for each path were calculated using JASP 0.18.3.0 with the bias-corrected percentile bootstrap method with 5000 repetitions. The significance level was set at *p* < 0.05 for all analyses, and the effect was considered significant if the 95% confidence interval did not include zero in the mediation analysis.

## Results

### Test for common method bias

In the current study, self-reported measures were used, which can introduce common method bias due to specific items, scale types, and response formats [41]. This bias threatens the validity of conclusions about the relationships between the measurements. To assess common method bias, we used Harman’s one-factor test, a widely used technique for addressing this issue [41–45]. Specifically, we conducted an exploratory factor analysis on the variables in our study, opting for an unrotated factor solution. The analysis yielded that the first factor explaining the highest covariance at 27.54%. This finding indicates that the influence of common method bias on our study results is unlikely to be substantial.

### Preliminary analyses

We first analyzed each subscale for outliers and removed the outliers based on the box plot results. Then, we analyzed these variables for skewness, kurtosis, and normality using the Kolmogorov-Smirnov test (see Table 1). The Kolmogorov-Smirnov test for each variable yielded a *p*-value higher than the critical value of 0.05, indicating that these variables did not significantly deviate from the normal distribution.

**Table 1:** Skewness, kurtosis, normality test results, mean and standard deviation for each variable

Variable	Mean	SD	Skewness	Kurtosis	Kolmogorov-Smirnov
**Competitive anxiety**					
Somatic anxiety	1.74	0.52	0.56	-0.13	0.12
Worry	2.07	0.60	0.38	0.37	0.15
Concentration disruption	1.63	0.49	0.57	-0.10	0.12
**Need Satisfaction**					
Competence	4.59	0.77	0.77	0.19	0.14
Autonomy	4.39	0.70	0.89	0.90	0.16
Relatedness	4.81	0.89	0.57	-0.59	0.15
**Athlete Burnout**					
Emotional and physical exhaustion	2.58	0.73	-0.24	-0.35	0.14
Reduced achievement	2.92	0.44	0.11	0.43	0.16
Sport devaluation	2.61	0.68	-0.28	-0.18	0.13

Note: SD = Standard Deviation

### Correlation analysis

Subsequently, correlation analyses were conducted to examine the relationships between the variables. Detailed results can be found in Table 2.

**Table 2:** Correlation analysis of variables in this study (*N* = 685, M ± SD)

Variable	1	2	3	4	5	6	7	8	9
1 Somatic	-								
2 Worry	0.70^**^	-							
3 Concentration	0.70^**^	0.62^**^	-						
4 ABQ R	0.22^**^	0.22^**^	0.30^**^	-					
5 ABQ E	0.37^**^	0.30^**^	0.42^**^	0.60^**^	-				
6 ABQ S	0.34^**^	0.26^**^	0.40^**^	0.61^**^	0.81^**^	-			
7 Competence	-0.25^**^	-0.27^**^	-0.30^**^	-0.24^**^	-0.51^**^	-0.43^**^	-		
8 Autonomy	-0.26^**^	-0.29^**^	-0.29^**^	-0.26^**^	-0.48^**^	-0.46^**^	0.71^**^	-	
9 Relatedness	-0.22^**^	-0.23^**^	-0.27^**^	-0.26^**^	-0.43^**^	-0.39^**^	0.70^**^	0.66^**^	-

Note: **p < 0 0.01; Somatic = somatic anxiety, Concentration = concentration disruption; ABQ R = ABQ reduced sense of accomplishment; ABQ E = ABQ emotional and physical exhaustion; ABQ S = sport devaluation; **Correlation is statistically significant at the 0.01 level (two tailed)

Correlation analyses revealed that somatic anxiety had a small correlation with reduced sense of accomplishment (*r* = 0.22, *p* < 0.01), and moderate correlations with emotional and physical exhaustion (*r* = 0.37, *p* < 0.01) and sport devaluation (*r* = 0.34, *p* < 0.01). Worry, on the other hand, had a small correlation with reduced sense of accomplishment (*r* = 0.22, *p* < 0.01) and sport devaluation (*r* = 0.26, *p* < 0.01), and a moderate correlation with emotional and physical exhaustion (*r* = 0.30, *p* < 0.01). Concentration disruption was moderately associated with reduced sense of accomplishment (*r* = 0.30, *p* < 0.01), emotional and physical exhaustion (*r* = 0.42, *p* < 0.01), and sport devaluation (*r* = 0.40, *p* < 0.01). In addition, somatic anxiety (correlation coefficients ranging from − 0.22 to -0.26) and worry (correlation coefficients ranging from − 0.23 to -0.29) both had small negative correlations with competence, autonomy, and relatedness. Concentration disruption has a moderate correlation with competence (*r*=-0.30, *p* < 0.01) and a small correlation with autonomy (*r*=-0.29, *p* < 0.01) and relatedness (*r*=-0.27, *p* < 0.01). Furthermore, competence had a small correlation with reduced sense of accomplishment (*r* = 0.24, *p* < 0.01), a large correlation with emotional and physical exhaustion (*r* = 0.51, *p* < 0.01), and a moderate correlation with sport devaluation (*r* = 0.43, *p* < 0.01). Finally, autonomy and relatedness each had a small correlation with reduced sense of accomplishment and moderate correlations with emotional exhaustion and sport devaluation, respectively.

### Regression analysis

Consistent with previous research, the current study found significant differences in competitive anxiety across gender and sport type. Gender showed significant differences in somatic anxiety (*t* = 2.69, *df* = 616, *p* = 0.007) and worry (*t* = 2.61, *df* = 616, *p* = 0.009), while sport type showed significant differences in somatic anxiety (*t* = 2.73, *df* = 616, *p* = 0.006) and concentration disruption (*t* = 2.14, *df* = 616, *p* = 0.032). Therefore, both variables were used as control variables in the subsequent regression and mediation analyses. To test hypotheses 1 and 2 of the current study, we conducted regression analyses in two stages (see Table 3). First, we examined the relationships between the three dimensions of competitive anxiety as independent variables and the three need satisfactions as dependent variables. Subsequently, we analyzed the associations between the dimensions of competitive anxiety and the need satisfactions (independent variables) in relation to total athlete burnout (dependent variable).

**Table 3:** Regression analysis results

Dependent variable	Independent variable	$\:\beta\:$	t	*R*	*R* ^2^	F
Competence	Somatic	-0.001	-0.01	0.32	0.10	13.77
	Worry	-0.149	-2.70			
	Concentration	-0.200	-3.59			
	Sport type	0.022	0.56			
	Gender	0.006	0.15			
Autonomy	Somatic	-0.034	-0.56	0.33	0.11	15.20
	Worry	-0.179	-3.25			
	Concentration	-0.154	-2.76			
	Sport type	0.019	0.49			
	Gender	0.088	2.27			
Relatedness	Somatic	-0.020	-0.32	0.29	0.09	11.62
	Worry	-0.101	-1.82			
	Concentration	-0.196	-3.48			
	Sport type	0.021	0.55			
	Gender	0.082	2.09			
Athlete burnout	Somatic	0.116	2.21	0.59	0.35	40.19
	Worry	-0.068	-1.43			
	Concentration	0.259	5.35			
	Competence	-0.178	-3.39			
	Autonomy	-0.208	-4.17			
	Relatedness	-0.074	-1.51			
	Sport type	-0.013	-0.39			
	Gender	-0.024	-0.70			

The regression results indicated that both worry ($\:\beta\:$=-0.149, *t*=-2.70, *p* = 0.007) and concentration disruption ($\:\beta\:$=-0.200, *t*=-3.59, *p* < 0.001) were negatively associated with competence. Worry ($\:\beta\:$=-0.179, *t*=-3.25, *p* = 0.001) and concentration disruption ($\:\beta\:$=-0.154, *t*=-2.76, *p* = 0.006) were also negatively correlated with autonomy. Only concentration disruption in competitive anxiety was negatively correlated with relatedness ($\:\beta\:$=-0.196, *t*=-3.48, *p* = 0.001). In addition, the results found that somatic anxiety ($\:\beta\:$=0.116, *t* = 2.21, *p* = 0.028) and concentration disruption ($\:\beta\:$=0.259, *t* = 5.35, *p* < 0.001) in competitive anxiety had a significant positive correlation with total athlete burnout scores. Since worry in competitive anxiety was not significantly correlated with athlete burnout, this result only partially supported Hypothesis 1. Finally, the regression results found that only competence ($\:\beta\:$=-0.178, *t*=-3.39, *p* = 0.001) and autonomy ($\:\beta\:$=-0.208, *t*=-4.17, *p* < 0.001) need satisfaction had a significant negative correlation with total athlete burnout scores. Overall, the regression analyses found that certain dimensions of the independent variable, competitive anxiety, were correlated with the mediator variable, need satisfaction. Additionally, some dimensions of competitive anxiety and need satisfaction were correlated with the dependent variable, athlete burnout. Therefore, based on these regression results, we conducted a mediation analysis to test Hypothesis 2 of the current study.

### Mediation model analysis

**Fig. 1:**
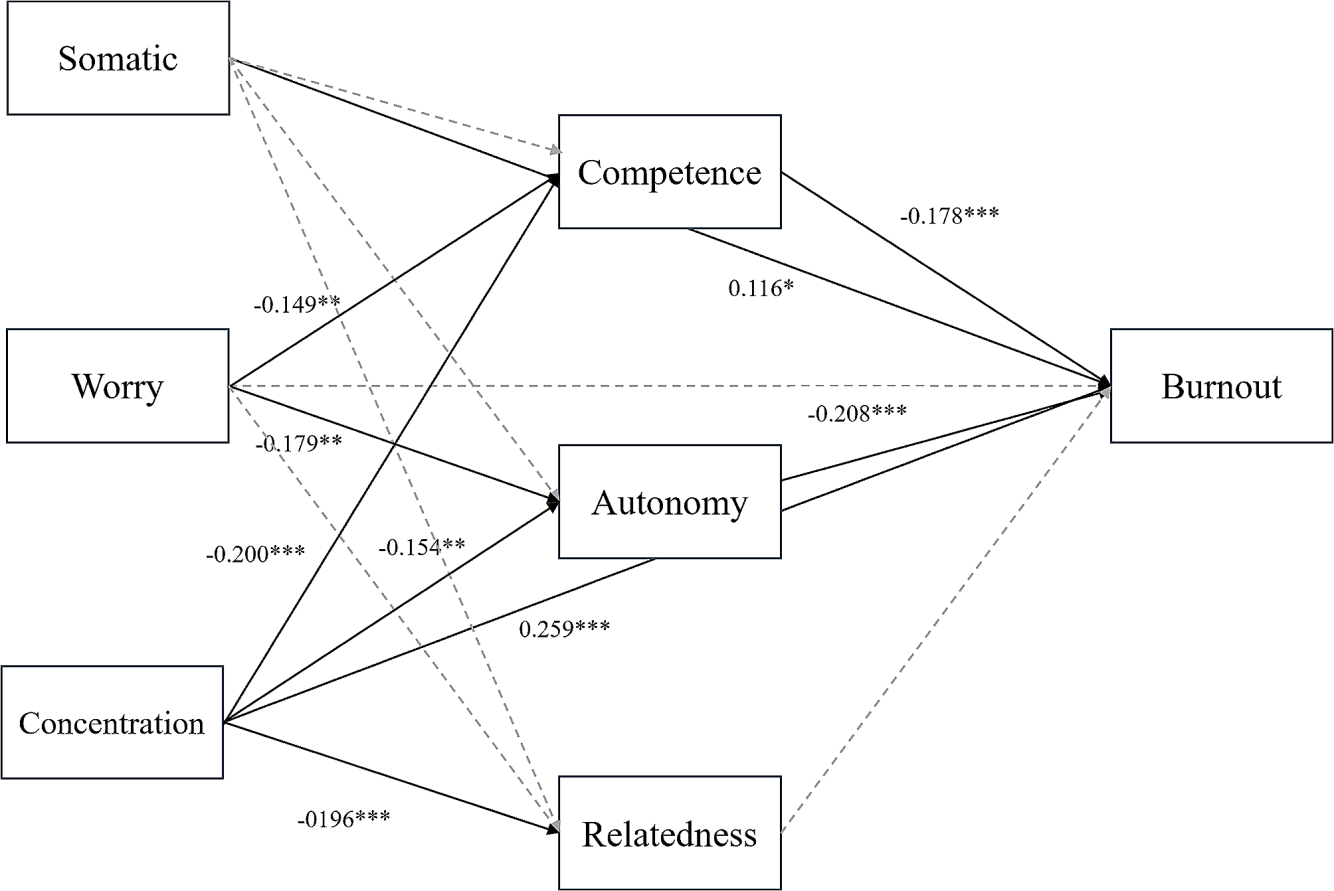
Path diagram illustrating the results of the mediation analyses. Notes: The grey broken line indicates a non-significant path effect, while the black solid line indicates a significant path effect. Only significant path effect statistics are provided for clarity in interpreting the results; Somatic = Somatic anxiety; Concentration = Concentration disruption; Burnout = Athlete burnout

In this mediation analysis, the three dimensions of competitive anxiety were the independent variables, the three needs satisfaction were the mediating variables, and athlete burnout was the dependent variable (See Fig. 1). Gender and sport type were included as control variables. The bootstrap bias-corrected percentile method was used for the mediation analysis with 5000 replications.

The mediation analyses revealed that the direct effects of somatic anxiety (Effect = 0.116, *p* = 0.026) and concentration disruption (Effect = 0.259, *p* < 0.001) were significant in relation to athlete burnout (See Table 4). Among the indirect effects, worry (Effect = 0.071, *p* = 0.002) and concentration disruption (Effect = 0.082, *p* < 0.001) were significantly related to athlete burnout. Finally, only somatic anxiety (Effect = 0.124, *p* = 0.031) and concentration disruption (Effect = 0.341, *p* < 0.001) had a significant total effect in relation to athlete burnout. The results also partially support Hypothesis 2, indicating that competence and autonomy in need satisfaction mediate the relationship between competitive anxiety (worry and concentration disruption) and athlete burnout.

**Table 4:** Effects of each path

Path	Estimate	SE	z-value	*p*	95% Confidence Interval	
					Lower	Upper
***Direct effects***						
Somatic - Burnout	0.116	0.052	2.221	0.026	0.009	0.222
Worry - Burnout	-0.068	0.047	-1.438	0.151	-0.157	0.024
Concentration - Burnout	0.259	0.048	5.389	< 0.001	0.164	0.357
***Total Indirect effects***						
Somatic - Burnout	0.009	0.025	0.345	0.730	-0.041	0.054
Worry - Burnout	0.071	0.023	3.047	0.002	0.027	0.118
Concentration - Burnout	0.082	0.024	3.430	< 0.001	0.040	0.129
***Total effects***						
Somatic - Burnout	0.124	0.058	2.153	0.031	0.013	0.234
Worry - Burnout	0.003	0.052	0.061	0.951	-0.089	0.101
Concentration - Burnout	0.341	0.053	6.477	< 0.001	0.245	0.444

## Discussion

Using a cross-sectional design, this study comprehensively examined the relationship between competitive anxiety and athlete burnout, focusing on the potential mediating role of the three need satisfactions from a SDT perspective. It was found that somatic anxiety and concentration disruption in competitive anxiety were positively correlated with athlete burnout. Additionally, competence and autonomy in need satisfaction mediated the relationship between competitive anxiety (worry and concentration disruption) and athlete burnout. This study not only provides new evidence on the relationship between competitive anxiety and athlete burnout but also uncovers the characteristics of the relationship between different dimensions of competitive anxiety and athlete burnout. Most importantly, the mediating roles of competence and autonomy in need satisfaction were identified in the relationship between competition anxiety and athlete burnout.

The first aim of this study was to investigate the relationship between different dimensions of competitive anxiety and athlete burnout. The correlation analyses revealed that all three dimensions of competitive anxiety were positively correlated with all three dimensions of athlete burnout to varying degrees. These findings are consistent with the results of two previous studies [6, 21]. In subsequent regression analyses, we found that only somatic anxiety and concentration disruption were significantly associated with athlete burnout. Somatic anxiety assessed over-activated physiological factors, while concentration disruption assessed difficulties in focusing on aspects of the competitive event. These findings suggest that the component of anxiety related to physiological factors, rather than worry about performance, is more strongly related to athlete burnout. This finding aligns with the cognitive-affective model of athlete burnout, which posits that physiological responses, such as anxiety, occur when athletes perceive a situation as threatening during the dynamic development of burnout. Therefore, this study not only provides new evidence for understanding the relationship between competitive anxiety and athlete burnout but also further supports the cognitive-affective model of athlete burnout.

The second aim of this study was to explore the potential mediating role of need satisfaction in the relationship between competitive anxiety and athlete burnout. The findings indicated that only competence and autonomy in need satisfaction mediated the relationship between worry and concentration disruption with athlete burnout, respectively. This result is partially consistent with previous findings [29, 34], such as a study that found competence and relatedness mediates the relationship between social physique anxiety and physical activity [34]. In regression analyses, we found that the worry factor of competitive anxiety was not correlated with athlete burnout. However, mediation analyses revealed that competence and autonomy played a mediating role in the relationship between worry and athlete burnout. As mentioned above, worry involves evaluating concerns about underperformance and its negative consequences, specifically regarding self-ability and performance [17]. Competence refers to an individual’s feeling of capability and the opportunity to develop and demonstrate their talents in interaction with the environment [24]. Autonomy, on the other hand, pertains to an individual’s perception that their actions are determined by their own will and self. These concepts shed light on why competence and autonomy mediate the relationship between competitive anxiety (specifically worry and concentration disruption) and athlete burnout.

Despite previous research demonstrating the mediating roles of psychological need satisfaction in perfectionism and athlete burnout [30], and cognitive reappraisal in the relationship between competition anxiety and athlete burnout [35], no study has explored the mediating role of need satisfaction in competitive anxiety and athlete burnout. The findings of the current study not only contribute new evidence regarding the relationship between competitive anxiety and athlete burnout but also offer insights from SDT to illuminate this relationship. Given the cross-sectional design of the current study, caution should be taken in making causal inferences about the relationship between competitive anxiety and athlete burnout. Therefore, future studies employing diverse methods and designs are necessary to further explore the relationship between these two variables and gain deeper insights into understanding athlete burnout.

### Limitations and future research

The present study has certain limitations. Firstly, the current study used a cross-sectional design, which limits the ability to establish a causal relationship between competitive anxiety and athlete burnout. This design also does not provide insight into the dynamic effects of competitive anxiety on athlete burnout. For example, athletes may experience increased anxiety before and during competition, which may decrease after the competition ends. Therefore, future studies could adopt a cross-lagged design that combines multiple measures (e.g., self-report and galvanic skin indicators) to assess participants’ levels of anxiety and athlete burnout before, during, and after competition [46–49]. This approach could better clarify the relationship between competitive anxiety and athlete burnout. Secondly, the study’s sample comprised college athletes rather than elite athletes. This choice might limit the generalizability of the results. Previous research has identified substantial differences in facilitative anxiety level ratings between elite and sub-elite athletes [50]. Therefore, future research should explore the relationship between competitive anxiety and burnout among elite athletes to better understand this relationship across different sample characteristics [51].

## Conclusions

Overall, this study investigated the relationship between competitive anxiety and athlete burnout, as well as the mediating role of need satisfaction, using a cross-sectional design among college athletes. The results showed that concentration disruption and somatic anxiety in competitive anxiety were positively related to athlete burnout. In addition, competence and autonomy in need satisfaction mediated the relationship between worry as well as concentration disruption in competitive anxiety and athlete burnout. These findings provide new insights into the relationship between competitive anxiety and athlete burnout.

## Data Availability

The data from this study has been uploaded to the Open Science Framework (OSF) and a specific access address will be opened after the article is accepted for publication.
